# Contactless Monitoring of Breathing Pattern and Thoracoabdominal Asynchronies in Preterm Infants Using Depth Cameras: A Feasibility Study

**DOI:** 10.1109/JTEHM.2022.3159997

**Published:** 2022-03-21

**Authors:** Valeria Ottaviani, Chiara Veneroni, Raffaele L. Dellaca’, Anna Lavizzari, Fabio Mosca, Emanuela Zannin

**Affiliations:** Technologies for Respiration Laboratory—TechRes LabDepartment of Electronic, Information and Bioengineering (DEIB)Politecnico di Milano University 20133 Milan Italy; NICUFondazione IRCCS Ca’ Granda Ospedale Maggiore Policlinico 20122 Milan Italy; Department of Clinical Sciences and Community HealthUniversity of Milan 9304 20122 Milan Italy; NICUFondazione Monza e Brianza per il Bambino e la sua Mamma 20900 Monza Italy

**Keywords:** Breathing pattern, depth camera, newborn infants, respiratory movements, RGB-D sensors, thoracoabdominal displacements

## Abstract

Objective: Monitoring infants’ breathing activity is crucial in research and clinical applications but remains a challenge. This study aims to develop a contactless method to monitor breathing patterns and thoracoabdominal asynchronies in infants inside the incubator, using depth cameras. Methods: We proposed an algorithm to extract the 3D displacements of the ribcage and abdomen from the analysis of depth images. We evaluated the accuracy of the system *in-vitro* vs. a reference motion capture analyzer. We also conducted a feasibility study on 12 patients receiving non-invasive respiratory support to estimate the mean and the variability of the chest wall displacements in preterm infants and evaluate the suitability of the proposed system in the clinical setting. Results: *In-vitro*, the mean (95% CI) error in the measurement of amplitude, frequency and phase shift between compartmental displacements was −0.14 (−0.57, 0.28) mm, 0.02 (−0.99, 1.03) bpm, and −0.40 (−1.76, 0.95)°, respectively. *In-vivo*, the mean (95% CI) amplitude of the ribcage and abdomen displacements were 0.99 (0.34, 2.67) mm and 1.20 (0.40, 2.15) mm, respectively. Conclusions: The developed system proved accurate *in-vitro* and was suitable for the clinical environment. Clinical Impact: The proposed method has value for evaluating infants’ breathing patterns in research applications and, after further development, may represent a simple monitoring tool for infants’ respiratory activity inside the incubator.

## I. Introduction

The assessment of breathing pattern and thoracoabdominal asynchronies (*e.g*., the non-synchronous motion of the ribcage and abdomen) in infants is crucial in clinical practice and research applications to evaluate control of breathing and respiratory distress in response to physiological conditions (*e.g.*, sleep), pharmacological treatments (*e.g.*, caffeine or surfactant), and respiratory support strategies. The standard way to assess infant breathing patterns is by a pneumotachograph connected to a face mask [1]. This approach has several limitations: i) the mask introduces dead space and alters breathing patterns [2], [3]; ii) leaks are difficult to avoid and affect the measurements; iii) it does not provide information about thoracoabdominal asynchronies. For all these reasons, several methods for evaluating chest wall volume have been proposed.

Impedance pneumography uses skin electrodes (*e.g.*, ECG electrodes) to measure changes in the thoracic electrical impedance associated with changes in aeration and, therefore, breathing. This method is limited to respiratory rate monitoring in the neonatal intensive care unit (NICU). Contact methods for the measurement for chest wall movements include respiratory inductive plethysmography [4], optoelectronic plethysmography [5], [6], fiber optic plethysmography [7], and accelerometers [8]. Such methods may cause discomfort, particularly in preterm infants with fragile skin, who often have other devices attached to the thorax (*e.g.*, ECG electrodes, temperature probes). Structured light plethysmography is a contactless method that may overcome the abovementioned limitations [9] but is cumbersome and unsuitable for infants inside an incubator. Due to recent advances in camera technology and computer vision, researchers have developed compact and cheap contactless solutions to monitor respiratory activity [10]–[19]. Some of these solutions were designed for gating [14], [16] and polysomnographic [11], [17] applications and were validated in healthy adults [12], [14]–[17] or children [11], [13]. The infants’ small breathing movements and irregular breathing patterns limit the use of these tools in the NICU. Additional constraints are the limited distance between the camera and infant and the lack of light inside the incubator. Standard video cameras combined with advanced image processing techniques (*e.g.*, Photoplethysmography and motion magnification) have been used to monitor respiratory frequency in infants [10], [18], [19]. However, clinicians also evaluate other parameters - like the amplitude of chest wall movements, thoracoabdominal asynchronies, and distortion - to assess respiratory distress. Compact contactless methods are still lacking to assess such parameters inside the incubator.

This study aims to develop a contactless method based on small RGB and depth (RGB-D) sensors, to monitor chest wall displacements and thoracoabdominal asynchronies in preterm infants. Specifically, we developed an algorithm to reconstruct the 3D displacements of selected chest wall regions, which does not require to perfectly align the z-axis of the depth camera to the direction of motion of the subject’s chest wall surface. This allows the patients to change position without affecting the measurements, nor the use of markers or the camera’s calibration. We evaluated the accuracy of the proposed system *in-vitro*. We also ran a feasibility clinical study to evaluate the mean and the variability of the chest wall displacements in preterm infants and the applicability of the system to the NICU environment.

## II. Methods and Procedures

### A. Acquisition System

The acquisition system consists of factory calibrated Intel® RealSense™ Depth Cameras D415 (CAM system), which capture three simultaneous streams of data: color (RGB), infrared (IR), and depth (D) images. The depth measurement is based on the structured light technique. The depth resolution is up to $1280 \times 720$, the depth frame rate is up to 90 frames per second, and the minimum depth distance is 0.16 m. The maximum frame rate and the minimum depth distance depend on the resolution. The cameras are small, lightweight (99 mm $\times \,\, 20$ mm $\times \,\, 23$ mm, 72 g), and cheap (about 300 € each).

**FIGURE 1.**
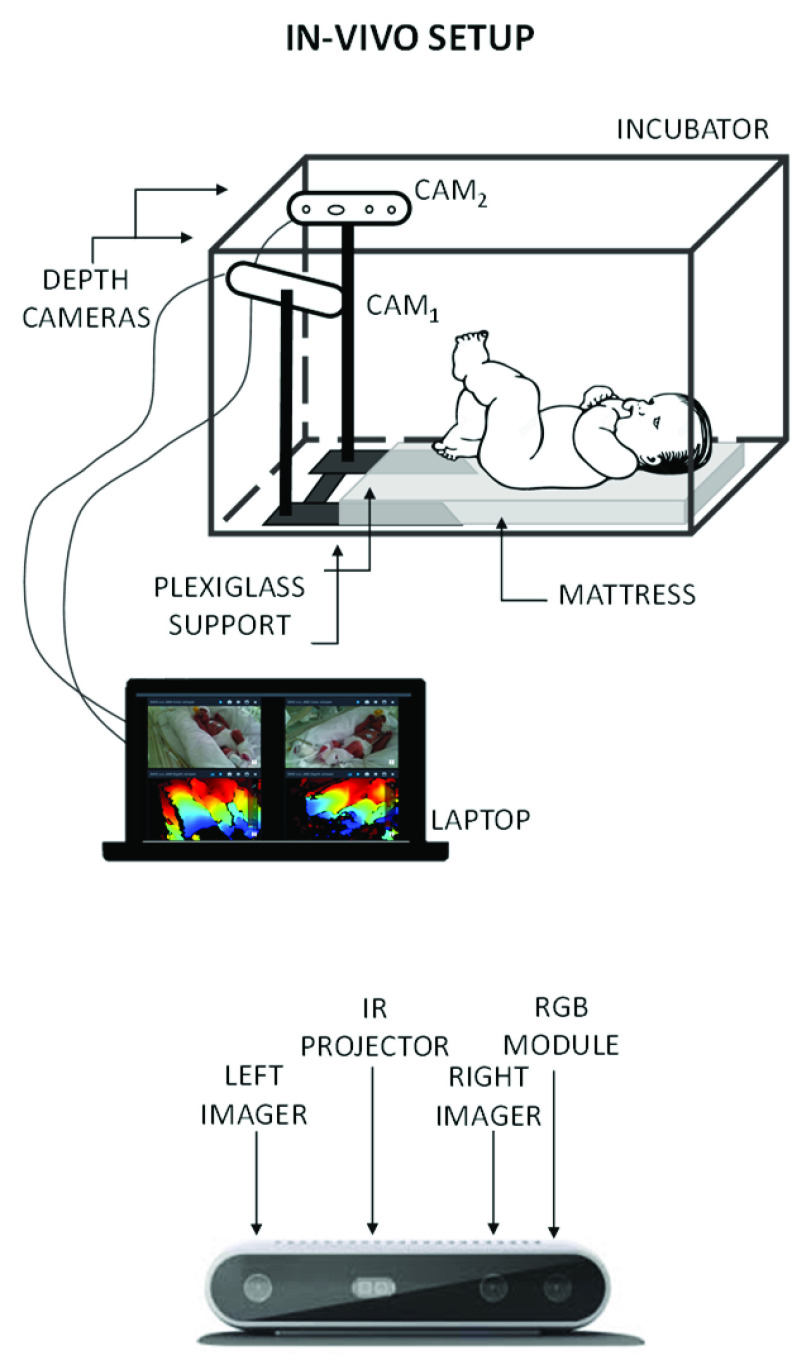
Proposed system for the assessment of thoraco-abdominal movements in new-borns. Top: arrangement of the measurement system with respect to the incubator. Bottom: Intel® RealSense™ Depth Cameras D415.

We captured infants’ chest wall movement from two viewpoints to reduce the risk of vision occlusion. The two RGB-D sensors were placed inside the incubator close to the infant’s feet, 35 cm apart and at an adjustable height of 25 to 30 cm. A U-shaped self-made plexiglass structure was used to secure and stabilize the position of the sensors inside the incubator (Fig. 1).

We used a depth resolution of $640 \times 480$ and a frame rate of 30 frames per second. Depth and color images were captured using the Intel® RealSense™ Viewer and saved on a personal computer in. *bag* file format for offline analysis.

### B. Data Processing

The RGB-D data streams were pre-processed to get a point cloud. The point cloud was further processed, using a specifically designed approach, to compute the 3D displacements of two user-selected chest wall points (one on the rib cage and one on the abdomen).

Our approach considered a 3D ROI around each of the two chest wall points, which we defined as a sphere with the lowest radius that allowed achieving clean average depth signals based on preliminary data. We hypothesized that the rib cage and chest wall surface included in the ROIs were small enough to be approximated to a plane, which we identified by linear regression. Finally, to estimate the 3D displacements of the rib cage and abdomen points belonging to the defined ROIs, we defined two cylinders having the ROIs as bases and the normals to the planes as principal axes. We hypothesized that with such an approach, we could get reproducible measurements of the 3D displacements of the selected points regardless of the orientation of the depth cameras

**FIGURE 2.**
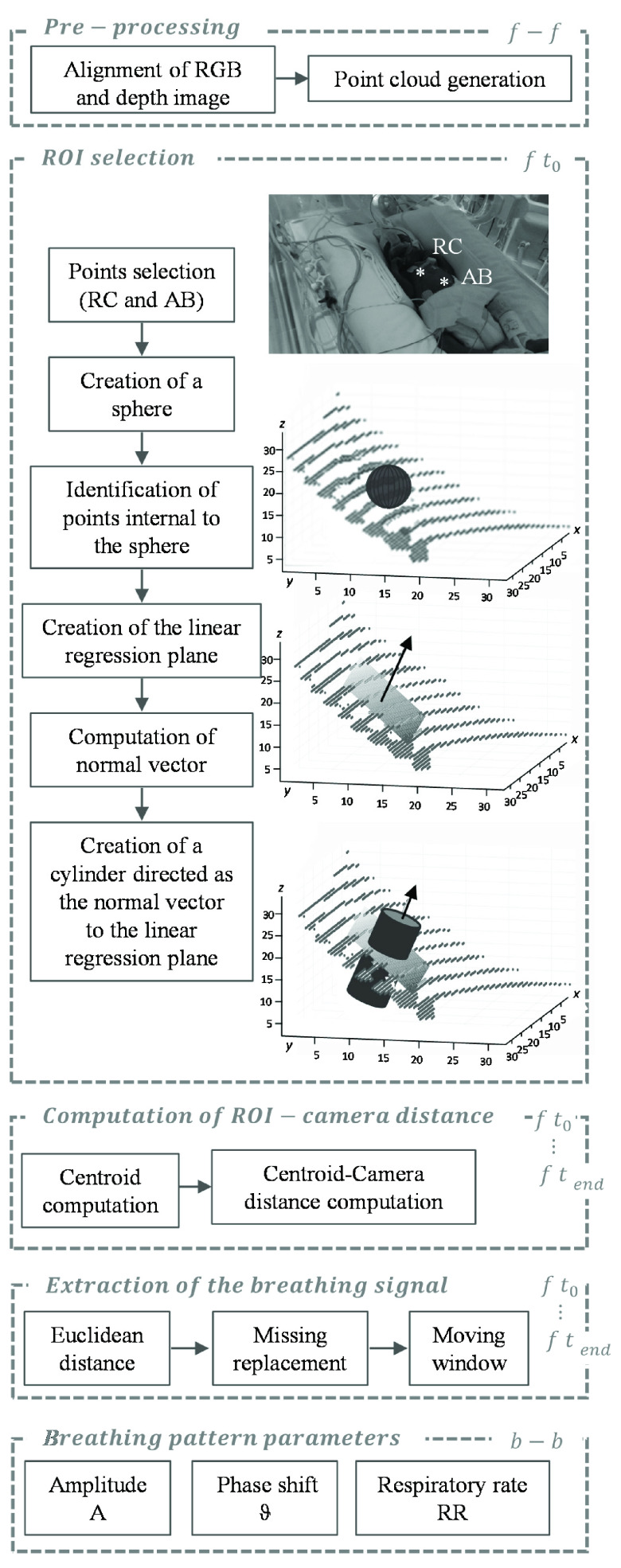
Flow chart of the algorithm developed for the extraction of breathing pattern parameters from the RGB-D images, with a focus on the novel approach proposed in this study. $f - f$: operation performed for each frame; $b - b$: operation performed for each breath; $f~\text{t}_{0}$: fist frame; $f~\text{t}_{\mathrm {end}}$: last frame. XYZ axis are expressed in mm.

The proposed algorithm is illustrated in Fig. 2 and was implemented using Matlab 2020b (MathWorks, Natick, MA, USA).

#### 1) Pre-Processing of the Raw Data

We applied the following pre-processing steps to the data from each sensor, taking advantage of the RealSense™ software development kit (SDK 2.0) wrapper functions:

• Alignment of the depth and color images: we used the *align* wrapper function, which applies extrinsic calibration parameters to the RGB and depth images to perform the spatial alignment of data streams captured from different viewpoints;
• Generation of the point clouds: we used the *point cloud* wrapper function of the Real Sense Point Cloud Library to convert the depth data stream into a point cloud.

We used the RGB data to select reference chest wall points on the first frame. Thanks to the spatial alignment between the RGB and depth images, we identified the points of the point cloud corresponding to the points selected by the user and applied all the following processing steps to the ROIs.

#### 2) ROI Selection on the Thorax and Abdomen

• Manual selection of one point on each compartment ( $\text{x}_{\mathrm {c}}$, $\text{y}_{\mathrm {c}}$);
• Generation of a sphere of 8-pixel radius centred in ( $\text{x}_{\mathrm {c}}$, $\text{y}_{\mathrm {c}}$);
• Identification of data points within the sphere;
• Identification of the linear regression plan;
• Computation of the vector normal to the regression plane;
• Generation of a cylinder of 8-pixel radius, with the center in ( $\text{x}_{\mathrm {c}}$, $\text{y}_{\mathrm {c}}$) and principal axis normal to the regression plane.

#### 3) Computation of ROI-Camera Distance

For each frame $i$, the algorithm automatically computed:

• The ROI: points inside the cylinder;
• The centroid: mean of the points inside the ROI ( $\text{x}_{\mathrm {mean}}$, $\text{y}_{\mathrm {mean}}$, $\text{z}_{\mathrm {mean}}$).
• The distance between centroid and camera P( $i$).

#### 4) Extraction of the Breathing Signal

We extracted the breathing signal for each chest wall compartment as the 3D displacement of the point selected by the operator. Specifically, we applied the following steps:

• Identification of the minimum ROI-camera distance ( $\text{P}_{\mathrm {min}}$);
• Computation of the Euclidean distance between $\text{P}_{\mathrm {min}}$ and P( $i$);
• Replacement of missing values and outliers (displacements larger than 15 cm) by linear interpolation;
• Signal filtering by moving average over five samples.

Regarding the use of two depth cameras, we did not apply an environment-based calibration to refer the two depth images to a common reference system. We independently treated the data streams from each camera to extract two breathing signals for each sensor, one for the rib cage and one for the abdomen. We manually checked for possible artefacts (*e.g.*, due to vision occlusion) on each breathing signal. If the signals of corresponding compartments from the two sensors were free from obvious artefacts, we averaged them; otherwise, we considered the cleanest signal.

#### 5) Computation of Breathing Pattern Parameters

We automatically detected local maxima and minima of the breathing signal over 33 samples (30 ms) windows, and we defined a breath as a sequence of a minimum-maximum-minimum. For each breath, we computed the following parameters breath-by-breath:

• Respiratory rate (RR, bpm) was computed as the inverse of the intra-breath interval, the time interval between two consecutive minima defining a breath;
• Displacements amplitude (A, mm) was computed as the difference in amplitude between the maximum and the following minimum;
• Phase shift between abdominal and ribcage displacements ( $\vartheta $, °) from the Lissajous loops was obtained by plotting ribcage versus abdominal volume tracings [20]. The loop’s width is a measure of asynchrony between the ribcage and abdomen: when they are synchronous, the loop is closed; as asynchrony increases, the loop widens. $\vartheta $ ranges from 0° (synchronous) to ±180° (paradoxical breathing). Negative values indicate that the outward motion of the abdomen precedes that of the ribcage.

### C. $In-Vitro$ Validation

**FIGURE 3.**
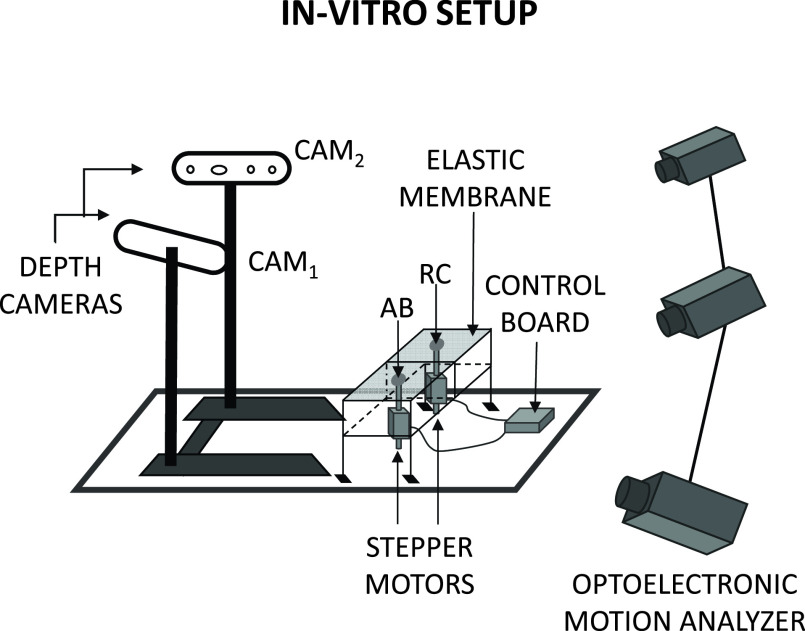
Experimental set-up used for the in-vitro validation. The set-up includes the proposed CAM system, the bi-compartmental test object with two points (RC and AB) moved by stepper motors driven by the control board.

We evaluated the system accuracy *in-vitro* against a commercial optoelectronic motion analyzer (OEP System, BTS, Milan, Italy), widely used for the measurement of respiratory movements [21]–[25]. The system captures the 3D displacement of reflective markers using eight infrared video cameras detecting flashing infrared light-emitting diodes. The images were captured at a frame rate of 100 Hz. At least two cameras should see each marker simultaneously to reconstruct their 3D position by stereo-photogrammetry. Fig. 3 shows the experimental setup used for *in-vitro* validation. The RGB-D sensors were positioned as described above (see *Acquisition system*).

We developed a bi-compartmental test object specifically designed to test the ability of the proposed method to reconstruct the displacements along the direction perpendicular to the object surface to challenge the algorithm’s ability to identify the movement direction and accurately estimate the 3D chest wall displacements even when the direction of the movement differs from the direction of the z-axis of the depth sensors. The test object consisted of a rectangular structure divided into two equal compartments representing the ribcage (RC) and abdomen (AB). The upper face of each compartment was a deformable membrane realized using a piece of elastic black synthetic fabric (polypropylene 97%, elastane 3%). Two stepper motors driven by an Arduino-based control board deformed the membranes to generate different breathing patterns.

The breathing pattern parameters chosen for the *in-vitro* validation spanned the ranges that we expect to encounter in neonates and infants:

• The amplitude was changed from 1 to 7 mm in steps of 1 mm, keeping phase shift and respiratory rate constant at 0° and 40 bpm, respectively;
• The phase shift was set to 0° (in-phase), 45°, and 90° (counter phase), keeping amplitude and respiratory rate constant at 3 mm and 40 bpm, respectively;
• The respiratory rate was set to 20, 40, and 60 bpm, keeping A and $\vartheta $ constant at 3mm and 0°, respectively.

**FIGURE 4.**
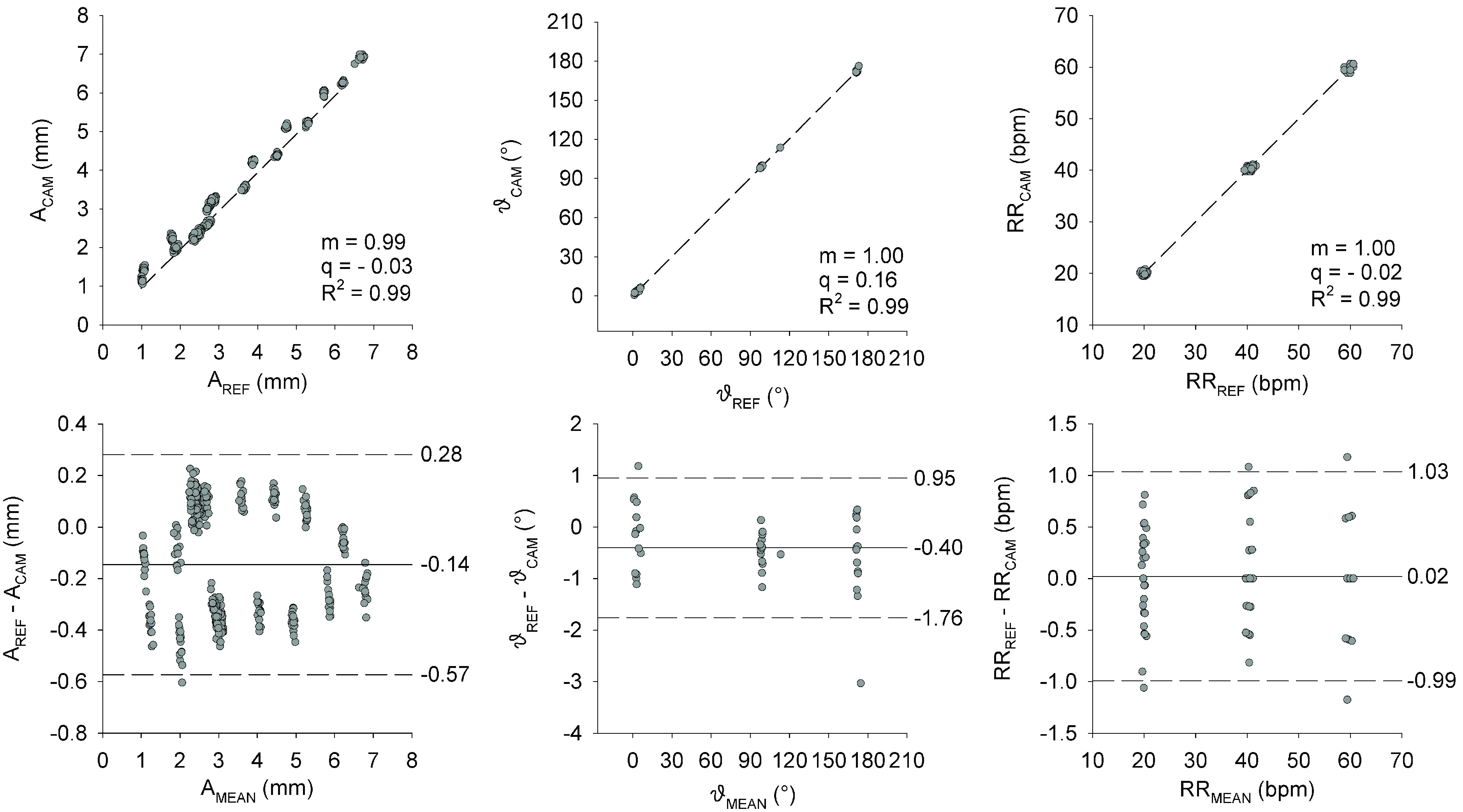
Upper panels: Relationship between breathing pattern parameters measured by the reference (REF) and the RealSense™ (CAM) systems. $\text{A}_{\mathrm {REF}}$ and $\text{A}_{\mathrm {CAM}}$: breath-by-breath amplitude of the breathing signal measured by REF and CAM. $\vartheta _{\mathrm {REF}}$ and $\vartheta _{\mathrm {CAM}}$: breath-by-breath phase shift of the breathing signal measured by REF and CAM. RR_REF_ and RR_CAM_: breath-by-breath frequency of the breathing signal measured by REF and CAM. The dashed line represents the regression line. Lower panels: Bland-Altman plots. Solid lines represent mean differences (bias), dashed lines represent the limits of agreement (mean difference ± 1.96 SD).

We analyzed 14 cycles for each condition. Consistently, the phase shift and frequency validations included 42 cycles (14 cycles $\times $ 3 protocol steps) each. The amplitude validation included seven steps with 14 cycles each plus all cycles with an amplitude of 3 mm in which we changed phase shift (42 cycles) and frequency (42 cycles), leading to a total of 182 cycles included in the analysis. Finally, we measured the amplitude of two compartments for each protocol step, which were needed to compute the phase shift and were considered independently for the amplitude and frequency validation. In summary, the results reported below (Table 1 and Fig. 4) refer to 42, 82, and 364 data points for phase shift, respiratory rate, and amplitude, respectively.

**TABLE 1:** Displacement Amplitude Accuracy

$S$	A_REF_ (mm)	A_CAM_ (mm)	E_(REF–CAM)_ (mm)	E_(REF–CAM)_ (%)	|E_(REF–CAM)_| (mm)	|E_(REF–CAM)_| (%)
1	1.0(0.0)	1.3(0.2)	−0.3(0.1)	−23.9(12.7)	0.3(0.1)	23.9(12.7)
2	1.8(0.1)	2.1(0.1)	−0.3(0.2)	−14.8(11.2)	0.3(0.2)	14.9(11.2)
3	2.7(0.2)	2.8(0.4)	−0.1(0.2)	−4.4(8.5)	0.2(0.1)	8.3(4.7)
4	3.8(0.1)	3.9(0.3)	−0.1(0.2)	−2.7(6.0)	0.2(0.1)	5.8(2.9)
5	4.6(0.1)	4.8(0.4)	−0.1(0.2)	−2.6(5.3)	0.2(0.1)	5.1(2.7)
6	5.5(0.2)	5.6(0.4)	−0.1(0.2)	−1.8(3.2)	0.2(0.1)	3.1(2.0)
7	6.4(0.2)	6.6(0.3)	−0.1(0.1)	−2.2(1.5)	0.1(0.1)	2.2(1.5)

S: step number; A_REF_: amplitude measured using the REF system, A_CAM_: amplitude measured using the CAM system, E_(REF-CAM)_: the difference between A_REF_ and A_CAM_, E_(REF-CAM)_: percentage error calculated as ((A_REF_ - A_CAM_)/ A_REF_)*100; |E_(REF - CAM)_|: the absolute difference between A_REF_ and A_CAM_, |E_(REF - CAM)_|: percentage error calculated as (abs((A_REF_ - A_CAM_)/ A_REF_))*100. Data are reported in terms of mean (SD). S1, S2, S4-S7 include 14 cycles. S3 includes 98 cycles

We placed one reflective marker on each compartment to allow for motion capture by the reference system. We simultaneously measured the motion of the test object using our system and the reference system.

We analyzed the CAM system data as described above. To analyze the data from the reference system, we used the OEP Tracker to reconstruct the 3D coordinates of each marker frame-by-frame. We then applied the last two steps of the proposed algorithm to extract the breathing signal and compute breathing pattern parameters. We expressed the accuracy of amplitude displacements in terms of absolute and percentage errors. Finally, we compared the breathing pattern parameters measured by the two systems using Bland-Altman graphs and linear regressions.

### D. Clinical Study

We conducted a feasibility clinical study in the Neonatal Intensive Care Unit of Fondazione IRCCS Cà Granda, Ospedale Maggiore Policlinico. The study was approved by the local ethics committee (protocol number 160_2019bis), and written informed consent was obtained from the parents before enrolment. The study aimed to estimate the mean and the variability of the chest wall displacements in preterm infants. Such information is needed to optimize the design of the measurement system, calculate the sample size for a future full-scale clinical validation of the system, and evaluate the clinical applicability of the proposed system. We did not perform a formal sample size calculation, but we used the rule of thumb of 12 patients, which was considered adequate for our aim [26]. We recruited preterm infants greater than 750 g body weight, receiving non-invasive respiratory support and without major malformations. Measurements were performed with infants in the supine position with the thorax naked. Vital parameters were monitored throughout the study period.

Chest wall movements were captured during 5 minutes of quiet breathing. For each recording, we manually discarded data segments corrupted by evident artefacts (*e.g*., major patient movements, occlusion of both cameras by the operator). Then, we computed the breathing pattern parameters on a breath-by-breath basis, as described above, and reported the mean (SD) values.

## III. Results

### A. $In-Vitro$ Validation

Table 1 reports the amplitude of the displacements measured using the reference and CAM systems. Our system tended to overestimate small displacements. The mean (SD) error in the measurement of 1-mm displacements was −0.3 (0.1) mm, corresponding to −24 (13) %.

Fig. 4 shows the agreement between the breathing pattern parameters measured using the reference and CAM systems. The mean difference in amplitude, phase shift, and frequency were −0.15 mm, −0.40°, and −0.02 bpm, respectively. The Bland-Altman plots did not show any proportional bias, and the linear regression analyses confirmed an excellent agreement between the two systems.

### B. Clinical Study

The measurement system proved suitable for the NICU environment: it was easy to assemble and disassemble inside the incubator, did not interfere with clinical practice or with the other bedside instrumentation, and captured RBG-D data streams of adequate quality to extract the breathing signals.

Fig. 5 shows the ribcage and abdominal movements of a representative infant. The abdominal displacements were larger than the ribcage ones, and the two compartments were out of phase. We analyzed the breathing pattern of twelve infants, with a mean (SD) gestational age (GA) of 30.71 (2.59) weeks, post-menstrual age of 31.36 (2.33) weeks, and bodyweight of 1205 (355) g. Table 2 reports the breathing pattern parameters for each patient. The mean (95% CI) amplitude of ribcage and abdominal displacements were 0.99 (0.34, 2.67) and 1.20 (0.40, 2.15) mm, respectively. The mean (95% CI) respiratory rate was 66 (37, 96) bpm. The abdominal displacements were larger than the ribcage displacements in 8 infants (67%). The thoracoabdominal movements were asynchronous (phase shift > 90°) in 5 (42%) infants, and the outward displacement of the abdomen preceded the ribcage (phase shift < 0°) in 4 (33%) infants.

**TABLE 2:** Breathing Pattern Parameters

	# breaths	A_RC_ (mm)	A_AB_ (mm)	$\vartheta$ (°)	RR (bpm)
1	75	0.3(0.1)	1.0(0.2)	−14(9)	52(11)
2	21	2.9(0.4)	1.7(0.1)	+13(9)	98(7)
3	69	0.7(0.2)	1.5(0.5)	+16(8)	58(23)
4	44	1.6(1.3)	1.9(0.5)	+147(11)	54(7)
5	10	1.1(0.1)	0.8(0.1)	−12(8)	58(5)
6	20	0.5(0.1)	0.8(0.2)	+126(11)	91(11)
7	38	0.6(0.2)	0.8(0.3)	+10(7)	33(13)
8	27	0.6(0.1)	2.2(0.2)	+161(10)	75(12)
9	23	2.0(1.1)	1.9(0.9)	−30(19)	49(18)
10	33	0.7(0.1)	0.8(0.1)	+32(13)	80(12)
11	10	0.5(0.1)	0.6(0.1)	+130(42)	58(4)
12	44	0.6(0.1)	0.3(0.1)	−83(60)	84(26)

# breaths: Number of breaths included in the analysis, A_RC_: amplitude of the ribcage displacement, $\text{A}_{\mathrm {AB}}$: amplitude of the abdominal displacement, $\vartheta$: phase shift between ribcage and abdomen displacements, RR: Respiratory Rate. All data are reported as mean (SD).

**FIGURE 5.**
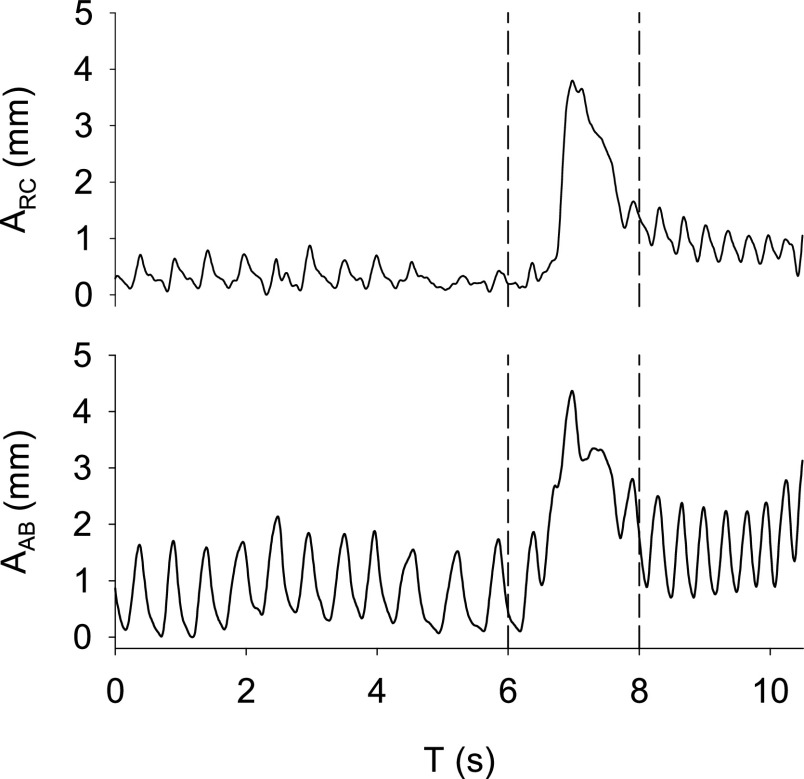
Representative tracings of ribcage (top panel) and abdominal (bottom panel) displacements. Vertical dashed lines define an example of data segments that were manually discarded due to movement artefacts.

## IV. Discussion

The assessment of breathing pattern and thoracoabdominal asynchronies in preterm infants is crucial to evaluate the maturity and stability of respiratory control, identify respiratory distress, and tailor the respiratory support strategy. However, measuring respiratory activity in infants remains a challenge. This study presents a compact, cheap, and contactless method based on two RGB-D cameras suitable for assessing breathing patterns and thoracoabdominal asynchronies in preterm infants.

The novelty of the proposed approach is that it reconstructs the 3D displacement of selected points of the chest wall surface even if the camera is not in front of the subject, without using markers and without calibrating the position of the camera with respect to the subject’s reference system.

The proposed approach monitors the movements of selected points on the rib cage and abdominal compartments. Dividing the chest wall into such two compartments is very common in clinical practice and physiological studies [27] because thoracoabdominal asynchronies and paradoxical movements are well-recognized signs of respiratory distress signs. Several parameters are used in the literature to quantify the relative contribution of the rib cage and abdominal compartments to total chest wall volume changes, thoracoabdominal asynchronies, and paradoxical breathing. However, the assumption that the chest wall has only two degrees of freedom is a simplification that may not hold in case of high degrees of chest wall distortion, which may occur in neonates with severe respiratory distress due to thehighly compliant chest wall.

The proposed system proved accurate *in-vitro,* measuring displacements with a mean error of −0.14 mm. There are no published guidelines or requirements for the accuracy of chest wall displacements or thoracoabdominal asynchronies. The feasibility study allows for the comparison of the accuracy observed *in-vitro* with typical values in the target population. In the smallest infants, chest wall displacements were less than 1 mm, particularly in the ribcage compartment. Such displacements are quite small compared to the limits of agreement (−0.57; 0.25 mm) estimated *in-vitro*. This result does not invalidate the appropriateness of the proposed method for the reconstruction of the 3D displacements of selected chest wall points but suggests that RGB-D sensors with higher depth resolution (*e.g.*, Intel® RealSense™ D415) may be preferable for very small infants (*e.g.,* less than 1000 g). Finally, the feasibility study also showed that the measurement setup was suitable for the clinical environment. It fitted inside the incubator, did not interfere with the clinical practice or other bedside instrumentation, and captured RGB-D images of adequate quality to extract the respiratory signal.

### A. Comparison With Other Studies

With the advances in cameras technology and computer vision, researchers developed different methods for assessing respiratory activity based on cheap and compact RBG-D sensors [28] (Table 3). One approach, suitable for gating applications, uses markers [29] or amplification surfaces [16]. However, the use of markers in contact with the skin is not ideal for neonatal applications. An alternative, contactless approach evaluates changes of the depth signals in specific regions of interest on the chest wall [9], [12], [14], [15], [30]. This method works well if the subject sits or lays in front of the camera; otherwise, only the movement in the depth axis direction is captured, reducing the measurement’s sensitivity. However, the incubator imposes constraints on the camera’s position relative to the subject, and the tiny infant’s respiratory movements require high sensitivity. A third approach consists of triangulating the thoracoabdominal surface and calculating the volume within this closed surface. This approach uses multiple cameras at different view angles to create 3D representations of the subject’s thoracoabdominal surface, build a 3D mesh, and calculate the volume at each frame [13], [17]. The use of multiple cameras implies the need for system calibration to identify the transformation matrices that allow for the alignment of the point clouds generated by each device. The advantage is the possibility to compute total and compartmental volume changes without a subject-specific calibration. The disadvantage for neonatal applications is the need for a high spatial view of the thoracoabdominal surface, which may be prevented by the nappy and the cloths used for nesting and swaddling the baby, and the incubator’s constraints.

**TABLE 3:** Comparison Between Different Methods to Evaluate Chest Wall Volume Changes

Technique	Working principle	Parameters measured	Reference	Applied to infants	Contactless	Advantages	Disadvantages
Structured light pletysmography	A structured light pattern is projected on the chest, the movement of the grid are viewed by two digital cameras. A 3D model allows for the computation of the volume enclosed within the chest wall surface.	Respiratory rate, compartmental chest wall volume changes, thoracoadbominal asynchronies	[9]	Yes	Yes	No need for calibration, contactless/no markers needed	Cumebrsome and expensive hardware, need to keep the infant in the supine position, need for a high spatial view.
RGB-D sensors + depth in ROIs	Compact RGB-D sensors measure changes in depth in specific chest wall ROIs	Respiratory rate	[9], [12], [14], [15], [30]	No	Yes	Cheap, compact, contactless	Only movements in the direction of the camera are detected.
RGB-D sensors + thorax segmentation	Depth data are used to reconstruct a 3D mesh of the chest wall and the volume enclosed within the surface is estimated.	Respiratory rate, compartmental chest wall volume changes, thoracoadbominal asynchronies	[13], [17]	No	Yes	Cheap, compact, contactless	Need to keep the infant in the supine position, need for a high spatial view.
RGB-D sensors + marker	Use of RGB and depth images to track the 3D position of a marker.	Respiratory rate, chest wall movements	[16], [29]	No	No	Measures 3D displacement of anatomical reperes, suitable for gating applications	Need for markers.
Video camera	Analysis of RGB images (e.g. by PhotoPlethysmography and motion magnification).	Respiratory rate	[10], [18], [19]	Yes	Yes	Contactless, use of standard cameras	Only vital signs are monitored.
Proposed method	Use of 2 RGB-D sensors + algorithm for reconstruction of 3D displacements of selected points of the rib cage and abdomen.	Respiratory rate, compartmental chest wall movements, thoracoadbominal asynchronies			Yes	Cheap, compact, contactless, no need for high spacial view, no need for specific relative position between camera and patient	Susceptible to motion artifacts and vision occlusion

Finally, researchers have also used standard video cameras combined with advanced image processing algorithms to monitor infants inside the incubator [10], [18], [19]. The advantage of this approach is that no special equipment is needed; the disadvantage is that only breathing frequency can be measured, while thoracoabdominal movements and asynchronies are clinically relevant signs of respiratory distress.

### B. Critiques of the Technique

Advantages of the proposed method include that the system fits inside the incubator; it does not need markers in contact with the skin; it works in low-lit environments; the infant’s chest wall does not need to be perpendicular to the z-axis of the depth camera; it does not need system calibration; it works well even when only part of the thoracoabdominal surface is visible.

The proposed method has some limitations. First, body movements and visual occlusion (*e.g.*, as the baby moves the limbs) could affect the measurement accuracy. Second, the infant needs to be in the supine position with a naked chest. Third, the user must manually select one point on the ribcage and one on the abdomen, which may be impractical in the clinical setting. Finally, the current version of the algorithm works offline and is not for real-time monitoring. Future developments of the algorithm may include the real-time analysis of the RGB-D data streams and the automatic identification of the ROIs, for example, by facial localization followed by morphological operations, from the position of skeletal joints, or body edge or image skeleton extraction followed by respiratory region estimation [9].

### C. Strengths and Limitations of the Study

Strengths of the study include a realistic test object able to reproduce several different thoracoabdominal motion patterns and a very accurate reference system for the *in-vitro* validation. The main limitation is the limited number of patients in the clinical study and the lack of a reference system for assessing the accuracy of chest wall displacements and thoracoabdominal asynchronies in infants.

## V. Conclusion

The present study proposes a contactless method based on cheap and compact RBG-D sensors to assess thoracoabdominal movements and asynchronies in infants. The measurement system can fit inside the incubator, and the proposed data processing algorithm allows computing the 3D displacements of chest wall points even if the camera is not in front of the infant. We evaluated the method’s accuracy vs. a high-resolution motion capture analyzer on a bi-compartmental test object, accurately replicating infants’ ribcage and abdomen movements. The system proved able to monitor 1-mm displacements with a mean error of −0.14 mm and was in good agreement with the reference system. Finally, the system proved suitable for the clinical environment.
